# Design of ANCHOR-RA: a multi-national cross-sectional study on screening for interstitial lung disease in patients with rheumatoid arthritis

**DOI:** 10.1186/s41927-024-00389-4

**Published:** 2024-05-21

**Authors:** Jeffrey A. Sparks, Philippe Dieudé, Anna-Maria Hoffmann-Vold, Gerd R Burmester, Simon LF Walsh, Michael Kreuter, Christian Stock, Steven Sambevski, Margarida Alves, Paul Emery

**Affiliations:** 1 Division of Rheumatology, Inflammation, and Immunity, Brigham and Women’s Hospital, Harvard Medical School, 60 Fenwood Road, #6016U, Boston, MA 02115 USA; 2 Department of Rheumatology, Bichat-Claude Bernard University Hospital, Assistance Publique-Hôpitaux de Paris, INSERM UMR1152, University of Paris, Paris, France; 3 Department of Rheumatology, Oslo University Hospital, University of Zurich, Oslo, Norway; 4 Department of Rheumatology, University Hospital Zurich, University of Zurich, Zurich, Switzerland; 5 Department of Rheumatology and Clinical Immunology, Charité – Universitätsmedizin Berlin, Berlin, Germany; 6 National Heart and Lung Institute, Imperial College London, London, UK; 7 Center for Pulmonary Medicine, Departments of Pneumology, Critical Care & Sleep Medicine, Mainz University Medical Center and of Pulmonary, Marienhaus Clinic Mainz, Mainz, Germany; 8 Boehringer Ingelheim Pharma GmbH & Co. KG, Ingelheim am Rhein, Germany; 9 Boehringer Ingelheim International GmbH, Ingelheim am Rhein, Germany; 10 NIHR Leeds Biomedical Research Centre, Institute of Rheumatic and Musculoskeletal Medicine, Leeds Teaching Hospitals NHS Trust and Leeds, University of Leeds, Leeds, UK

**Keywords:** Connective tissue diseases, Pulmonary fibrosis, Rheumatic diseases, Tomography

## Abstract

**Background:**

Patients with rheumatoid arthritis (RA) are at risk of developing interstitial lung disease (ILD), which is associated with high mortality. Screening tools based on risk factors are needed to decide which patients with RA should be screened for ILD using high-resolution computed tomography (HRCT). The ANCHOR-RA study is a multi-national cross-sectional study that will develop a multivariable model for prediction of RA-ILD, which can be used to inform screening for RA-ILD in clinical practice.

**Methods:**

Investigators will enrol consecutive patients with RA who have ≥ 2 of the following risk factors for RA-ILD: male; current or previous smoker; age ≥ 60 years at RA diagnosis; high-positive rheumatoid factor and/or anti-cyclic citrullinated peptide (titre > 3 x upper limit of normal); presence or history of certain extra-articular manifestations of RA (vasculitis, Felty’s syndrome, secondary Sjögren’s syndrome, cutaneous rheumatoid nodules, serositis, and/or scleritis/uveitis); high RA disease activity in the prior 12 months. Patients previously identified as having ILD, or who have had a CT scan in the prior 2 years, will not be eligible. Participants will undergo an HRCT scan at their local site, which will be assessed centrally by two expert radiologists. Data will be collected prospectively on demographic and RA-related characteristics, patient-reported outcomes, comorbidities and pulmonary function. The primary outcomes will be the development of a probability score for RA-ILD, based on a multivariable model incorporating potential risk factors commonly assessed in clinical practice, and an estimate of the prevalence of RA-ILD in the study population. It is planned that 1200 participants will be enrolled at approximately 30 sites in the USA, UK, Germany, France, Italy, Spain.

**Discussion:**

Data from the ANCHOR-RA study will add to the body of evidence to support recommendations for screening for RA-ILD to improve detection of this important complication of RA and enable early intervention.

**Trial registration:**

clinicaltrials.gov NCT05855109 (submission date: 3 May 2023).

## Background

Patients with rheumatoid arthritis (RA) are at risk of developing interstitial lung disease (ILD). The reported prevalence of ILD in patients with RA varies widely depending on the population studied and the criteria used to define ILD, with estimates ranging between 2% and 41% [1–6]. RA-ILD confers a substantially increased risk of mortality [4, 7–11]. A recent meta-analysis of data from 15 studies in patients with RA-ILD found a mortality rate of 49% over 5 to 10 years [12]. Patients with RA may also have other respiratory complications such as pleural disease [13], bronchiectasis [6, 14] or emphysema [6, 15].

The high mortality associated with RA-ILD means that it is important that it is detected and treated promptly. Indeed, a delay in the diagnosis of RA-ILD has been shown to be an independent predictor of mortality [16]. Early detection of RA-ILD would require screening of asymptomatic patients, as substantial lung damage and loss of lung function may occur before patients report respiratory symptoms such as dyspnoea [17–19] and respiratory symptoms may be underestimated in patients in whom joint disease impairs exertion. However, in an international survey of 354 rheumatologists conducted in 2019, 44% did not consider screening for RA-ILD to be necessary in patients with risk factors but no respiratory symptoms [20].

The gold standard for radiological assessment of ILD is a high-resolution computed tomography (HRCT) scan [21, 22]. Evidence is emerging on the use of lung ultrasound as a screening tool [23–27], but more data are needed to establish its accuracy in the detection of RA-ILD. The high prevalence of RA and lower prevalence of RA-ILD means that it would be inefficient and likely not feasible for all patients with RA to undergo HRCT, but there is no consensus as to which patients should be screened. Many factors have been associated with the development of ILD in patients with RA, including older age, male sex, smoking history, seropositivity for rheumatoid factor (RF) or anti-cyclic citrullinated peptide (CCP), elevated erythrocyte sedimentation rate (ESR), moderate or high RA disease activity, and the *MUC5B* promoter variant [3, 7, 28–33]. The risk of ILD appears to be similar in patients with RA taking different types of disease-modifying anti-rheumatic drugs (DMARDs) [34], although recent analyses have suggested a lower risk in patients taking tofacitinib compared with adalimumab [35] and in patients taking abatacept plus methotrexate compared with methotrexate alone [36]. The extent to which treatments for RA modify the risk of ILD, and whether any reduction in risk is due to better control of RA disease activity or to specific effects in the lung, remains unclear.

The *A*utoantibodies, *N*on-articular manifestations of RA, *C*igarettes, *H*e/him, *O*lder age at RA onset, *R*A high disease activity (ANCHOR)-RA study is a multi-national cross-sectional study that will develop a multivariable model for prediction of RA-ILD, which can be used to inform screening for RA-ILD in clinical practice. This study will also evaluate the prevalence of RA-ILD, features of RA-ILD on HRCT, and other pulmonary complications of RA, in patients with risk factors for RA-ILD. A sub-study will compare the performance of lung ultrasound and HRCT in the detection of ILD. This manuscript describes the design of the ANCHOR-RA study.

## Methods/design

### Study design and participants

The ANCHOR-RA study is a cross-sectional study that will be conducted at approximately 30 sites in the USA, UK, Germany, France, Italy, Spain. Eligible patients will be diagnosed with RA according to the 1987 American College of Rheumatology (ACR) [37] or the 2010 ACR/European Alliance of Associations for Rheumatology (EULAR) [38] classification criteria, with any duration of RA, and have ≥ 2 of the following risk factors for RA-ILD: male; current or previous smoker; age ≥ 60 years at RA diagnosis; high-positive RF and/or anti-CCP (titre > 3 x upper limit of normal); presence or history of certain extra-articular manifestations of RA (vasculitis, Felty’s syndrome, secondary Sjögren’s syndrome, cutaneous rheumatoid nodules, serositis, and/or scleritis/uveitis); high RA disease activity in the prior 12 months (Table 1). Patients will be excluded if they have previously been identified as having ILD, have had a chest CT in the prior 2 years, have received treatments known to induce ILD (e.g. radiation therapy to chest, bleomycin), have had a lung transplant, or have been diagnosed with an autoimmune disorder overlapping with RA, except secondary Sjögren’s syndrome. There is no restriction on the use of disease-modifying antirheumatic drugs (DMARDs).

**Table 1:** Disease activity measures used to ascertain high RA disease activity in the ANCHOR-RA study

Measure	Range	High RA disease activity
PAS score [39]	0 to 10	≥ 8.0 to ≤ 10.0
PAS-II score [39]	0 to 10	≥ 8.0 to ≤ 10.0
RAPID-3 score [40]	0 to 10	> 4 to ≤ 10
CDAI score [41]	0 to 76	> 22.0
DAS-28-ESR or DAS-28-CRP [42, 43]	0 to 9.4	> 5.1
SDAI score [44]	0 to 86	> 26

CDAI, Clinical Disease Activity Index; DAS-28-ESR, Disease Activity Score with 28 joints using erythrocyte sedimentation rate; DAS-28-CRP, Disease Activity Score with 28 joints using C-reactive protein; PAS, Patient Activity Scale; RA, rheumatoid arthritis; RAPID-3, Routine Assessment of Patient Index Data 3; SDAI, Simple Disease Activity Index

Investigators will enrol consecutive patients from RA outpatient clinics using one of three methods: (1) they may contact consecutive patients from their clinic database to ascertain interest and screen them for eligibility; (2) they may conduct a search of their clinic database to identify patients who have ≥ 2 of the specified risk factors for RA-ILD and then contact consecutive patients in that group to ascertain interest and screen for eligibility, or (3) they may screen consecutive patients for eligibility and interest during routine clinic sessions.

The study will be carried out in compliance with the protocol, the principles laid down in the Declaration of Helsinki, and in accordance with the International Council for Harmonisation of Technical Requirements for Pharmaceuticals for Human Use (ICH), Harmonised Tripartite Guideline for Good Clinical Practice and Guidelines for Good Pharmacoepidemiology Practices. The Advarra Institutional Review Board has approved the study protocol (Pro00071380). All participants will provide written informed consent prior to entering the study. The study is registered on clinicaltrials.gov (NCT05855109; submission date: 3 May 2023).

### Data collection

Participants will undergo a volumetric HRCT scan using a standardised multi-detector row CT acquisition protocol at their local site. Visual assessment of scans will be conducted centrally by two expert radiologists. Imaging features on HRCT will be assessed according to established recommendations [21, 22, 45]. The presence/absence of ILD, free-standing bronchiectasis and emphysema will be adjudicated. Features related to these diseases will be assessed (Table 2). ILD will be defined as non-dependent abnormalities, including ground glass or reticular abnormalities, lung distortion, traction bronchiectasis, honeycombing, or non-emphysematous cysts. If there is disagreement between the radiologists regarding the presence or absence of ILD, bronchiectasis, or emphysema, a third expert radiologist will make the decision. HRCT scans will also be read by a local radiologist and incidental findings reported back to the investigator, as per standard of care.

**Table 2:** Features on HRCT that will be assessed in the ANCHOR-RA study

Features related to ILD	Presence or absence of ILD (adjudicated)
	Presence or absence of: ground glass opacities traction bronchiectasis reticulations honeycombing
	CT pattern: usual interstitial pneumonia (UIP) non-specific interstitial pneumonia (NSIP) (cellular/fibrotic subtypes) lymphocytic interstitial pneumonia organising pneumonia (OP) diffuse alveolar damage respiratory bronchiolitis desquamative interstitial pneumonia, indeterminate
	Distribution of ILD: upper, middle or lower lung regions, or diffuse
	Predominance of ILD: upper, middle or lower lung regions, or diffuse
	Total extent of fibrosis to nearest 10%
	Total extent of ILD to nearest 10%
**Features related to bronchiectasis**	Presence or absence of free-standing bronchiectasis (adjudicated)
	Lobes involved
	Presence or absence of: mucus plugging bronchial wall thickening
**Features related to emphysema**	Presence or absence of emphysema (adjudicated)
	Distribution of emphysema: upper, middle or lower lung regions, or diffuse
	Extent of emphysema to nearest 10%

HRCT, high-resolution computed tomography; ILD, interstitial lung disease

Data will be collected prospectively on demographic and RA-related characteristics (including disease activity based on Disease Activity Score with 28 joints [DAS-28]), extra-articular features, comorbidities, pulmonary function and patient-reported outcomes (Table 3). The investigator will be required to start these assessments within 90 days of the participants providing informed consent and collect the data within a 90-day period. Participants can opt in or out of *MUC5B* promoter variant testing at a central laboratory and of biobanking of DNA, plasma and serum samples for future analyses.

**Table 3:** Participant information to be collected prospectively in the ANCHOR-RA study

Demographics	Age
	Sex
	Height
	Weight
	Race/ethnicity
	Smoking status and pack/years
RA-related characteristics	Family history of RA
	Family history of ILD
	Duration of RA
	DAS-28
	Physician global assessment
	Presence of secondary Sjögren’s syndrome, Felty’s syndrome, vasculitis, ulcers, uveitis, scleritis, cutaneous rheumatoid nodules
	RA deformities such as ulnar deviation, subluxation, swan necking, boutonniere’s deformity
	Inflammatory markers (CRP, ESR)
Comorbidities and medications	Comorbidities
	Medications and doses
	Use of inhalants (never/past/current)
Respiratory-related assessments	Respiratory examination: presence of crackles, decreased breathe sounds, resting oxygen saturation, respiratory rate
	Spirometry (FVC in mL; FVC % predicted; FEV_1_ in mL; FEV_1_% predicted; FEV_1_/FVC ratio)
	DLco
	Environmental exposures (e.g. silica, asbestos)
	Previous COVID-19 infection
	HRCT (central read and local read)
Patient-reported outcomes (PROs)	RA-related PROs: MDHAQ [46] SF-36 [47] AA-VAS
	Lung-related PROs: mMRC dyspnoea scale [48] L-PF questionnaire [49] Presence of cough and dyspnoea: *“Do you have a cough that has been present for at least 8 weeks” (yes/no)* *“Do you have unusual shortness of breath that has been present for at least 8 weeks” (yes/no)* • Cough severity VAS (in participants who report cough)
Other	*MUC5B* promoter variant (for participants who opt in)
	DNA, plasma and serum biobanked samples (for participants who opt in)

The investigator will be required to start these assessments within 90 days of the participant providing informed consent and collect the data within a 90-day period

AA-VAS, Arthritis Activity Visual Analogue Scale; CRP, C-reactive protein; DAS-28, Disease Activity Score with 28 joints; DLco, diffusing capacity of the lung for carbon monoxide; ESR, erythrocyte sedimentation rate; FVC, forced vital capacity; FEV_1_, forced expiratory volume in one second; HRCT, high-resolution computed tomography; ILD, interstitial lung disease; L-PF, Living with Pulmonary Fibrosis; MDHAQ, multidimensional health assessment questionnaire; mMRC, modified Medical Research Council; RA, rheumatoid arthritis; SF-36, 36-Item short form survey; VAS, visual analogue scale

Data collected from medical records will comprise: age at RA diagnosis; previous treatments for RA; presence of erosions in hands on plain film radiography; disease activity based on the measures listed in Table 1 other than DAS-28 (remission/low/medium/high; first measures and last four measures); anti-SS-A/Ro antibody status; anti-SS-B/La autoantibody status; anti-nuclear antibody status and titres; RF status; anti-CCP status; and highest values and last four values for RF, anti-CCP, CRP and ESR.

### Outcomes

The primary outcomes will be the development of a probability score for RA-ILD, based on a multivariable model incorporating potential risk factors commonly assessed in clinical practice, and an estimate of the prevalence of RA-ILD and of radiological features of RA-ILD on HRCT in the study population.

Secondary outcomes will be the demographic and disease characteristics of participants with RA who do and do not have respiratory symptoms (cough and/or dyspnoea), the performance of the model in sub-populations with and without cough and/or dyspnoea, and correlations of pulmonary function tests and abnormal findings on auscultation with findings on HRCT.

Further outcomes include a multivariable model that additionally considers the *MUC5B* promoter variant as a potential predictor of RA-ILD. The performance of the model to predict RA-ILD defined using different extents of ILD and patterns on HRCT will be assessed in sensitivity analyses. The prevalence and HRCT features of free-standing bronchiectasis and emphysema will also be assessed.

### Model development and validation

Model development and validation will follow recommendations for diagnostic models [50]. Candidate risk factors for RA-ILD will be identified based on expert opinion, supported by a systematic literature search. A logistic regression model using least absolute shrinkage and selection operator (LASSO) penalisation will be fit with the full set of candidate predictors. The model may include a set of pre-specified terms to consider potential non-linear and interaction effects. The penalty parameter will be determined by a cross-validation procedure. The model will then be re-fit via classical maximum-likelihood methods with only those predictors with coefficients not set to zero in the first fit. Some predictors may be forced into the model following consultation with the steering committee. The overall performance of the model will be assessed using the Brier Score, Nagelkerke R² and adjusted Nagelkerke R². Discrimination will primarily be assessed using the area under the receiver operating characteristic (ROC) curve (c-statistic). Calibration will be assessed by a plot. Internal validation will be performed using a bootstrapping approach, which will be used to correct for over-optimism in the performance of the model. The ability of the model to identify participants who have RA-ILD on HRCT will be assessed by determining a cut-off for predicted probability (decision threshold). Participants with a predicted probability above the threshold will be classified as positive (i.e. as having RA-ILD) and participants with a predicted probability under the threshold as negative (i.e. as not having RA-ILD). Further performance measures (including sensitivity, specificity, positive and negative predictive values) of the model will be assessed. A similar process will be used to derive a model that additionally considers the *MUC5B* promoter variant. Reporting will follow the Transparent Reporting of a multivariable prediction model for Individual Prognosis or Diagnosis (TRIPOD) statement [51].

### Sample size calculation

It is planned that up to 1200 participants will be enrolled. This sample size was calculated based on an assumed number of model parameters of 22 to 24, an assumed prevalence of RA-ILD in the study population (which is enriched for patients with RA-ILD) of 30%, an assumed c-statistic for the model of 0.75, and an assumed shrinkage factor associated with the predictors of 0.9 [52]. In this setting, the number of events per predictor parameter would be 15. A difference of 0.05 between the apparent and adjusted R^2^ of the model and a margin of error of 0.05 in estimating the risk of RA-ILD when the coefficients for all candidate predictors are set to zero is expected. After HRCT results from the first 600 participants are available, an interim analysis will be performed to determine whether the prevalence of RA-ILD in the study population will enable the modelling based on risk factors or if the sample size needs to be adjusted.

### Ultrasound sub-study

To investigate the performance of lung ultrasound in detecting ILD compared to HRCT, including predictive values, sensitivity and specificity, a sub-study will be conducted at sites where rheumatologists have been trained on bedside lung ultrasound. A standardised approach to ultrasound probe, anatomical location and data collection will be provided. Images will be taken from 14 areas. The measures analysed will include the total number of B lines, pleural thickness (normal/abnormal), pleural regularity (normal/irregular) and the presence/absence of pleural effusion and consolidation. It is not intended that sites will refer participants to radiology for formal ultrasonographic assessment.

## Discussion

The data collected in the ANCHOR-RA study will establish the prevalence of RA-ILD in a high-risk population and enable development of a multivariable model for prediction of RA-ILD. This model will be based on risk factors that are easily assessed in clinical practice, which can be used to decide which patients with RA should be screened for ILD using HRCT. Risk scores developed in previous studies have shown good specificity and sensitivity for the identification of patients with RA-ILD [32, 53–57]. However, these risk scores were developed in small or single-centre populations [32, 53–56] and some were based on patients with known ILD [53, 57] or in patients referred for HRCT because ILD was suspected [56]. A risk score developed based on sex, smoking status, CDAI, ESR and extra-articular manifestations in 118 patients with RA had 90% sensitivity and 64% specificity for identifying patients with RA-ILD on HRCT [53], while a risk score developed based on sex, age at RA onset, DAS-28-ESR score and the *MUC5B* promoter variant in 163 patients with RA had 75% sensitivity and 85% specificity [32]. A risk score developed based on age at RA onset, smoking status, RF titre, CCP titre and DAS-28 score in 430 patients with RA had 86% sensitivity and 58% specificity for identifying patients with RA-ILD on HRCT [56]. A nomogram developed using data on sex, smoking status, RF, CRP and matrix metalloproteinase-3 from 223 patients at a single centre had a c-index of 0.826 for identification of RA-ILD compared with an assessment by a multidisciplinary team [55]. We seek to build on these studies by developing a risk score for RA-ILD that could easily be applied in clinical practice. The results of the ANCHOR-RA study will also elucidate the prevalence of RA-ILD in patients with particular risk factors. Understanding the prevalence of RA-ILD in sub-populations of patients with RA is important given its significant impact on prognosis [4, 7–11] and the burden that it places on healthcare resources [58].

The most frequent radiological patterns seen in patients with RA-ILD are usual interstitial pneumonia (UIP) and non-specific interstitial pneumonia (NSIP) [4, 59–61]. A UIP pattern on HRCT has been associated with worse survival in patients with RA-ILD [62–64]. Greater extents of fibrosis, traction bronchiectasis, honeycombing, and reticulation, and the presence of emphysema, have also been associated with worse survival [60, 64–66]. The ANCHOR-RA study will provide further data on the prevalence of these features on HRCT in patients with RA-ILD.

Early diagnosis of RA-ILD provides the opportunity to improve its monitoring and management. The course of RA-ILD is variable, with some patients experiencing rapid progression while others remain relatively stable [67, 68]. Progressive pulmonary fibrosis in patients with RA-ILD is characterised by increasing radiological fibrosis, decline in lung function, worsening symptoms, and early mortality [67, 69, 70]. Early detection of RA-ILD, and of its progression, enables treatment to be initiated promptly. Data from retrospective or uncontrolled studies suggest that immunosuppression may slow the progression of RA-ILD [71–74], but in the absence of randomised controlled trials, the effect of these therapies remains unclear. Based on the results of the randomised placebo-controlled INBUILD trial [75], the tyrosine kinase inhibitor nintedanib has been licensed for the treatment of progressive fibrosing ILDs of any aetiology and was given a conditional recommendation for use in patients with progressive pulmonary fibrosis who have failed standard management for fibrotic ILD in a clinical practice guideline [22]. Data from the subgroup of patients with progressive fibrosing RA-ILD in the INBUILD trial provide further support for its use in these patients [76].

Strengths of the ANCHOR-RA study include that it is large and multi-national, that consecutive recruitment of patients will minimise the risk of bias (e.g. investigators preferentially putting forward patients suspected to have ILD), and that a wide range of clinical and lung imaging data will be collected. Limitations include that the results will be applicable only to patients with RA who have the specified risk factors for ILD.

In conclusion, the results of the multi-national cross-sectional ANCHOR-RA study will enable the development of a probability score for RA-ILD based on a multivariable model incorporating risk factors and will elucidate the prevalence of RA-ILD in patients with multiple risk factors. These data will add to the body of evidence that will support recommendations for screening for RA-ILD to improve detection of this important complication of RA and enable early intervention.

## Data Availability

Not applicable (No data are provided in this manuscript).

## Abbreviations

AA-VAS: Arthritis Activity Visual Analogue Scale
ACR: American College of Rheumatology
CCP: cyclic citrullinated peptide
CDAI: Clinical Disease Activity Index
CRP: C-reactive protein
CT: computed tomography
DAS-28: Disease Activity Score with 28 joints
DAS-28-CRP: Disease Activity Score with 28 joints using C-reactive protein
DAS-28-ESR: Disease Activity Score with 28 joints using erythrocyte sedimentation rate
DLco: Diffusing capacity of the lung for carbon monoxide
DMARDs: Disease-modifying antirheumatic drugs
ESR: Erythrocyte sedimentation rate
EULAR: European alliance of associations for rheumatology
FEV< _1_: Forced expiratory volume in one second
FVC: Forced vital capacity
HRCT: High-resolution computed tomography
ILD: Interstitial lung disease
L-PF: Living with pulmonary fibrosis
MDHAQ: Multidimensional health assessment questionnaire
mMRC: modified Medical Research Council
NSIP: Non-specific interstitial pneumonia
PAS: Patient activity scale
RA: Rheumatoid arthritis
RAPID-3: Routine assessment of patient index data 3
RF: Rheumatoid factor
SDAI: Simple disease activity index
SF-36: 36-Item short form survey
UIP: Usual interstitial pneumonia
VAS: Visual analogue scale

## References

[1] Gabbay E, Tarala R, Will R, Carroll G, Adler B, Cameron D, Lake FR (1997). Interstitial lung disease in recent onset rheumatoid arthritis. Am J Respir Crit Care Med.

[2] Dawson JK, Fewins HE, Desmond J, Lynch MP, Graham DR (2001). Fibrosing alveolitis in patients with rheumatoid arthritis as assessed by high resolution computed tomography, chest radiography, and pulmonary function tests. Thorax.

[3] Doyle TJ, Patel AS, Hatabu H, Nishino M, Wu G, Osorio JC (2015). Detection of rheumatoid arthritis-interstitial lung disease is enhanced by serum biomarkers. Am J Respir Crit Care Med.

[4] Denis A, Henket M, Ernst M, Maes N, Thys M, Regnier C (2022). Progressive fibrosing interstitial lung disease in rheumatoid arthritis: a retrospective study. Front Med (Lausanne).

[5] Albrecht K, Strangfeld A, Marschall U, Callhoff J (2023). Interstitial lung disease in rheumatoid arthritis: incidence, prevalence and related drug prescriptions between 2007 and 2020. RMD Open.

[6] Hyldgaard C, Harders S, Blegvad J, Herly M, Masic D, Sofíudóttir BK, et al. Clinical and preclinical pulmonary disease in newly diagnosed rheumatoid arthritis: a two-year follow-up study. Scand J Rheumatol. 2023;1–8. 10.1080/03009742.2023.2194105. 10.1080/03009742.2023.2194105 37066633

[7] Bongartz T, Nannini C, Medina-Velasquez YF, Achenbach SJ, Crowson CS, Ryu JH (2010). Incidence and mortality of interstitial lung disease in rheumatoid arthritis: a population-based study. Arthritis Rheum.

[8] Olson AL, Swigris JJ, Sprunger DB, Fischer A, Fernandez-Perez ER, Solomon J (2011). Rheumatoid arthritis-interstitial lung disease-associated mortality. Am J Respir Crit Care Med.

[9] Hyldgaard C, Hilberg O, Pedersen AB, Ulrichsen SP, Løkke A, Bendstrup E, Ellingsen T (2017). A population-based cohort study of rheumatoid arthritis-associated interstitial lung disease: comorbidity and mortality. Ann Rheum Dis.

[10] Sparks JA, Jin Y, Cho SK, Vine S, Desai R, Doyle TJ, Kim SC (2021). Prevalence, incidence and cause-specific mortality of rheumatoid arthritis-associated interstitial lung disease among older rheumatoid arthritis patients. Rheumatology (Oxford).

[11] Samhouri BF, Vassallo R, Achenbach SJ, Kronzer VL, Davis JM, Myasoedova E, Crowson CS (2022). Incidence, risk factors, and mortality of clinical and subclinical rheumatoid arthritis-associated interstitial lung disease: a population-based cohort. Arthritis Care Res (Hoboken).

[12] Farquhar HJ, Beckert N, Beckert L, Edwards AL, Matteson EL, Frampton C (2023). Survival of adults with rheumatoid arthritis associated interstitial lung disease – a systematic review and meta-analysis. Semin Arthritis Rheum.

[13] Balbir-Gurman A, Yigla M, Nahir AM, Braun-Moscovici Y (2006). Rheumatoid pleural effusion. Semin Arthritis Rheum.

[14] Martin LW, Prisco LC, Huang W, McDermott G, Shadick NA, Doyle TJ, Sparks JA (2021). Prevalence and risk factors of bronchiectasis in rheumatoid arthritis: a systematic review and meta-analysis. Semin Arthritis Rheum.

[15] Jacob J, Song JW, Yoon HY, Cross G, Barnett J, Woo WL (2018). Prevalence and effects of emphysema in never-smokers with rheumatoid arthritis interstitial lung disease. EbioMedicine.

[16] Cano-Jiménez E, Vázquez Rodríguez T, Martín-Robles I, Castillo Villegas D, Juan García J, Bollo de Miguel E (2021). Diagnostic delay of associated interstitial lung disease increases mortality in rheumatoid arthritis. Sci Rep.

[17] Kanat F, Levendoglu F, Teke T (2007). Radiological and functional assessment of pulmonary involvement in the rheumatoid arthritis patients. Rheumatol Int.

[18] Gochuico BR, Avila NA, Chow CK, Novero LJ, Wu HP, Ren P (2008). Progressive preclinical interstitial lung disease in rheumatoid arthritis. Arch Intern Med.

[19] Chen J, Shi Y, Wang X, Huang H, Ascherman D (2013). Asymptomatic preclinical rheumatoid arthritis-associated interstitial lung disease. Clin Dev Immunol.

[20] Solomon JJ, Swigris JJ, Kreuter M, Polke M, Aronson K, Hoffmann-Vold AM, Dellaripa PF (2022). The attitudes and practices of physicians caring for patients with rheumatoid arthritis-associated interstitial lung disease: an international survey. Rheumatology (Oxford).

[21] Raghu G, Remy-Jardin M, Myers JL, Richeldi L, Ryerson CJ, Lederer DJ (2018). Diagnosis of idiopathic pulmonary fibrosis. An official ATS/ERS/JRS/ALAT clinical practice guideline. Am J Respir Crit Care Med.

[22] Raghu G, Remy-Jardin M, Richeldi L, Thomson CC, Inoue Y, Johkoh T (2022). Idiopathic pulmonary fibrosis (an update) and progressive pulmonary fibrosis in adults: an official ATS/ERS/JRS/ALAT clinical practice guideline. Am J Respir Crit Care Med.

[23] Moazedi-Fuerst FC, Kielhauser SM, Scheidl S, Tripolt NJ, Lutfi A, Yazdani-Biuki B (2014). Ultrasound screening for interstitial lung disease in rheumatoid arthritis. Clin Exp Rheumatol.

[24] Mena-Vázquez N, Jimenez-Núñez FG, Godoy-Navarrete FJ, Manrique-Arija S, Aguilar-Hurtado MC, Romero-Barco CM (2021). Utility of pulmonary ultrasound to identify interstitial lung disease in patients with rheumatoid arthritis. Clin Rheumatol.

[25] Wang Y, Chen S, Zheng S, Lin J, Hu S, Zhuang J (2021). The role of lung ultrasound B-lines and serum KL-6 in the screening and follow-up of rheumatoid arthritis patients for an identification of interstitial lung disease: review of the literature, proposal for a preliminary algorithm, and clinical application to cases. Arthritis Res Ther.

[26] Gutierrez M, Ruta S, Clavijo-Cornejo D, Fuentes-Moreno G, Reyes-Long S, Bertolazzi C (2022). The emerging role of ultrasound in detecting interstitial lung disease in patients with rheumatoid arthritis. Joint Bone Spine.

[27] Sofíudóttir BK, Harders SMW, Lage-Hansen PR, Christensen R, Munk HL, Sorensen GL (2022). Using thoracic ultrasound to detect interstitial lung disease in patients with rheumatoid arthritis: a protocol for the diagnostic test accuracy AURORA study. BMJ Open.

[28] Koduri G, Norton S, Young A, Cox N, Davies P, Devlin J (2010). Interstitial lung disease has a poor prognosis in rheumatoid arthritis: results from an inception cohort. Rheumatology (Oxford).

[29] Juge PA, Lee JS, Ebstein E, Furukawa H, Dobrinskikh E, Gazal S (2018). MUC5B promoter variant and rheumatoid arthritis with interstitial lung disease. N Engl J Med.

[30] Sparks JA, He X, Huang J, Fletcher EA, Zaccardelli A, Friedlander HM (2019). Rheumatoid arthritis disease activity predicting incident clinically apparent rheumatoid arthritis-associated interstitial lung disease: a prospective cohort study. Arthritis Rheumatol.

[31] Kronzer VL, Huang W, Dellaripa PF, Huang S, Feathers V, Lu B (2021). Lifestyle and clinical risk factors for incident rheumatoid arthritis-associated interstitial lung disease. J Rheumatol.

[32] Juge PA, Granger B, Debray MP, Ebstein E, Louis-Sidney F, Kedra J (2022). A risk score to detect subclinical rheumatoid arthritis-associated interstitial lung disease. Arthritis Rheumatol.

[33] Kim H, Cho SK, Song YJ, Kang J, Jeong SA, Kim HW (2023). Clinical characteristics of rheumatoid arthritis patients with interstitial lung disease: baseline data of a single-center prospective cohort. Arthritis Res Ther.

[34] Curtis JR, Sarsour K, Napalkov P, Costa LA, Schulman KL (2015). Incidence and complications of interstitial lung disease in users of tocilizumab, rituximab, abatacept and anti-tumor necrosis factor α agents, a retrospective cohort study. Arthritis Res Ther.

[35] Baker MC, Liu Y, Lu R, Lin J, Melehani J, Robinson WH (2023). Incidence of interstitial lung disease in patients with rheumatoid arthritis treated with biologic and targeted synthetic disease-modifying antirheumatic drugs. JAMA Netw Open.

[36] Dieudé P, Sparks J, Fischer A, Chen L, Lozenski K, Dahan S (2023). Incidence rates of interstitial lung disease among patients with rheumatoid arthritis treated with abatacept: a post hoc pooled analysis from a compendium of clinical trials. Ann Rheum Dis.

[37] Arnett FC, Edworthy SM, Bloch DA, McShane DJ, Fries JF, Cooper NS (1988). The American Rheumatism Association 1987 revised criteria for the classification of rheumatoid arthritis. Arthritis Rheum.

[38] Aletaha D, Neogi T, Silman AJ, Funovits J, Felson DT, Bingham CO (2010). 2010 rheumatoid arthritis classification criteria: an American College of Rheumatology/European League against Rheumatism collaborative initiative. Ann Rheum Dis.

[39] Wolfe F, Michaud K, Pincus T (2005). A composite disease activity scale for clinical practice, observational studies, and clinical trials: the patient activity scale (PAS/PAS-II). J Rheumatol.

[40] Pincus T, Swearingen CJ, Bergman M, Yazici Y (2008). RAPID3 (Routine Assessment of Patient Index Data 3), a rheumatoid arthritis index without formal joint counts for routine care: proposed severity categories compared to disease activity score and clinical disease activity index categories. J Rheumatol.

[41] Aletaha D, Nell VP, Stamm T, Uffmann M, Pflugbeil S, Machold K, Smolen JS (2005). Acute phase reactants add little to composite disease activity indices for rheumatoid arthritis: validation of a clinical activity score. Arthritis Res Ther.

[42] Prevoo ML, van’t Hof MA, Kuper HH, van Leeuwen MA, van de Putte LB, van Riel PL (1995). Modified disease activity scores that include twenty-eight‐joint counts. Development and validation in a prospective longitudinal study of patients with rheumatoid arthritis. Arthritis Rheum.

[43] Fransen J, Welsing P, de Keijzer R, van Riel P (2003). Disease activity scores using C-reactive protein: CRP may replace ESR in the assessment of RA disease activity. Ann Rheum Dis.

[44] Smolen JS, Breedveld FC, Schiff MH, Kalden JR, Emery P, Eberl G (2003). A simplified disease activity index for rheumatoid arthritis for use in clinical practice. Rheumatology (Oxford).

[45] Lynch DA, Sverzellati N, Travis WD, Brown KK, Colby TV, Galvin JR (2018). Diagnostic criteria for idiopathic pulmonary fibrosis: a Fleischner Society White Paper. Lancet Respir Med.

[46] Pincus T, Swearingen C, Wolfe F (1999). Toward a multidimensional Health Assessment Questionnaire (MDHAQ): assessment of advanced activities of daily living and psychological status in the patient-friendly health assessment questionnaire format. Arthritis Rheum.

[47] QualityMetric Incorporated. The SF-36v2^®^ Health Survey. https://www.qualitymetric.com/health-surveys/the-sf-36v2-health-survey. Accessed 16 June 2023.

[48] Medical Research Council. Modified Medical Research Council (mMRC) dyspnoea scale. Version developed in 1986. https://www.ukri.org/councils/mrc/facilities-and-resources/find-an-mrc-facility-or-resource/mrc-dyspnoea-scale. Accessed 16 June 2023.

[49] Swigris J, Cutts K, Male N, Baldwin M, Rohr KB, Bushnell DM (2021). The living with pulmonary fibrosis questionnaire in progressive fibrosing interstitial lung disease. ERJ Open Res.

[50] Steyerberg EW (2019). Clinical prediction models. A practical approach to development, validation, and updating.

[51] Collins GS, Reitsma JB, Altman DG, Moons KGM (2015). Transparent reporting of a multivariable prediction model for individual prognosis or diagnosis (TRIPOD): the TRIPOD statement. Ann Intern Med.

[52] Riley RD, Ensor J, Snell KIE, Harrell FE, Martin GP, Reitsma JB (2020). Calculating the sample size required for developing a clinical prediction model. BMJ.

[53] Paulin F, Doyle TJ, Mercado JF, Fassola L, Fernández M, Caro F (2021). Development of a risk indicator score for the identification of interstitial lung disease in patients with rheumatoid arthritis. Reumatol Clin (Engl Ed).

[54] Narváez J, Aburto M, Seoane-Mato D, Bonilla G, Acosta O, Candelas G (2023). Screening criteria for interstitial lung disease associated to rheumatoid arthritis: expert proposal based on Delphi methodology. Reumatol Clin (Engl Ed).

[55] Xue J, Hu W, Wu S, Wang J, Chi S, Liu X (2022). Development of a risk nomogram model for identifying interstitial lung disease in patients with rheumatoid arthritis. Front Immunol.

[56] Koduri GM, Podlasek A, Pattapola S, Zhang J, Laila D, Nandagudi A (2023). Four-factor risk score for the prediction of interstitial lung disease in rheumatoid arthritis. Rheumatol Int.

[57] Wheeler AM, Baker JF, Riley T, Yang Y, Roul P, Wysham KD, et al. Development and internal validation of a clinical and genetic risk score for rheumatoid arthritis-associated interstitial lung disease. Rheumatology (Oxford). 2024;keae001. 10.1093/rheumatology/keae001. 10.1093/rheumatology/keae001 PMC11701303 38243706

[58] Raimundo K, Solomon JJ, Olson AL, Kong AM, Cole AL, Fischer A, Swigris JJ (2019). Rheumatoid arthritis-interstitial lung disease in the United States: prevalence, incidence, and healthcare costs and mortality. J Rheumatol.

[59] Tanaka N, Kim JS, Newell JD, Brown KK, Cool CD, Meehan R (2004). Rheumatoid arthritis-related lung diseases: CT findings. Radiology.

[60] Nurmi HM, Kettunen HP, Suoranta SK, Purokivi MK, Kärkkäinen MS, Selander TA (2018). Several high-resolution computed tomography findings associate with survival and clinical features in rheumatoid arthritis-associated interstitial lung disease. Respir Med.

[61] Salaffi F, Carotti M, Di Carlo M, Tardella M, Giovagnoni A (2019). High-resolution computed tomography of the lung in patients with rheumatoid arthritis: prevalence of interstitial lung disease involvement and determinants of abnormalities. Med (Baltim).

[62] Solomon JJ, Chung JH, Cosgrove GP, Demoruelle MK, Fernandez-Perez ER, Fischer A (2016). Predictors of mortality in rheumatoid arthritis-associated interstitial lung disease. Eur Respir J.

[63] Solomon JJ, Ryu JH, Tazelaar HD, Myers JL, Tuder R, Cool CD (2013). Fibrosing interstitial pneumonia predicts survival in patients with rheumatoid arthritis-associated interstitial lung disease (RA-ILD). Respir Med.

[64] Kim HC, Lee JS, Lee EY, Ha YJ, Chae EJ, Han M (2020). Risk prediction model in rheumatoid arthritis-associated interstitial lung disease. Respirology.

[65] Kim EJ, Elicker BM, Maldonado F, Webb WR, Ryu JH, Van Uden JH (2010). Usual interstitial pneumonia in rheumatoid arthritis-associated interstitial lung disease. Eur Respir J.

[66] Oh JH, Kim GHJ, Cross G, Barnett J, Jacob J, Hong S, Song JW (2022). Automated quantification system predicts survival in rheumatoid arthritis-associated interstitial lung disease. Rheumatology (Oxford).

[67] Hyldgaard C, Ellingsen T, Hilberg O, Bendstrup E (2019). Rheumatoid arthritis-associated interstitial lung disease: clinical characteristics and predictors of mortality. Respiration.

[68] Chang SH, Lee JS, Ha YJ, Kim MU, Park CH, Lee JS (2023). Lung function trajectory of rheumatoid arthritis-associated interstitial lung disease. Rheumatology (Oxford).

[69] Zamora-Legoff JA, Krause ML, Crowson CS, Ryu JH, Matteson EL (2017). Progressive decline of lung function in rheumatoid arthritis-associated interstitial lung disease. Arthritis Rheumatol.

[70] Mena-Vázquez N, Rojas-Gimenez M, Romero-Barco CM, Manrique-Arija S, Francisco E, Aguilar-Hurtado MC (2021). Predictors of progression and mortality in patients with prevalent rheumatoid arthritis and interstitial lung disease: a prospective cohort study. J Clin Med.

[71] Fernández-Díaz C, Castañeda S, Melero-González RB, Ortiz-Sanjuán F, Juan-Mas A, Carrasco-Cubero C (2020). Abatacept in interstitial lung disease associated with rheumatoid arthritis: national multicenter study of 263 patients. Rheumatology (Oxford).

[72] Narváez J, Robles-Pérez A, Molina-Molina M, Vicens-Zygmunt V, Luburich P, Yañez MA (2020). Real-world clinical effectiveness of rituximab rescue therapy in patients with progressive rheumatoid arthritis-related interstitial lung disease. Semin Arthritis Rheum.

[73] Kim K, Woo A, Park Y, Yong SH, Lee SH, Lee SH (2022). Protective effect of methotrexate on lung function and mortality in rheumatoid arthritis-related interstitial lung disease: a retrospective cohort study. Ther Adv Respir Dis.

[74] Matson SM, Baqir M, Moua T, Marll M, Kent J, Iannazzo NS (2023). Treatment outcomes for rheumatoid arthritis-associated interstitial lung disease: a real-world, multisite study of the impact of immunosuppression on pulmonary function trajectory. Chest.

[75] Flaherty KR, Wells AU, Cottin V, Devaraj A, Walsh SLF, Inoue Y (2019). Nintedanib in progressive fibrosing interstitial lung diseases. N Engl J Med.

[76] Matteson EL, Aringer M, Burmester GR, Mueller H, Moros L, Kolb M (2023). Effect of nintedanib in patients with progressive pulmonary fibrosis associated with rheumatoid arthritis: data from the INBUILD trial. Clin Rheumatol.

